# Collection of cerebrospinal fluid into EDTA versus plain tubes does not affect the standard analysis in dogs

**DOI:** 10.1186/s13028-019-0457-1

**Published:** 2019-05-06

**Authors:** Bodil Cathrine Koch, Lea Ophelia Daniels, Line Tang Thomsen, Michelle Brønniche Møller Nielsen, Mette Berendt, Hanne Gredal

**Affiliations:** University Hospital for Companion Animals, Department of Veterinary Clinical Sciences, Faculty of Health and Medical Sciences, University of Copenhagen, Dyrlægevej 16, 1870 Frederiksberg C, Denmark

**Keywords:** CNS, CSF, Ethylenediaminetetraacetic acid, Inflammation, Laboratory results, Sample preservation

## Abstract

**Background:**

Cerebrospinal fluid (CSF) can be collected into ethylenediaminetetraacetic acid (EDTA) or plain tubes. The EDTA content presumably contributes to a better cell preservation. EDTA, however, is reported to cause a false elevation in the total protein concentration and to dilute the CSF sample, thereby affecting the diagnostic interpretation. To the authors’ knowledge, no validated studies support this view. The aim of this study was therefore to determine if the choice of tube (EDTA or plain) influences the results of the standard CSF analysis.

**Results:**

Thirty-two paired EDTA stabilised and plain CSF samples were included. There was no statistically significant difference in the semi-quantitative protein concentrations when comparing CSF samples from EDTA and plain plastic tubes (P > 0.99). The total nucleated cell count did not differ significantly between EDTA and plain tube samples (P = 0.85). There were no significant differences in the differential cell counts between the two tubes when evaluating polymorphonuclear cells (P = 0.90), lymphocytes (P = 0.84) and monocytes/macrophages (P = 0.86). Also, there was no significant difference in the preservation of cell morphology when evaluating cytological preparations from EDTA stabilised and plain tube samples (P = 0.45).

**Conclusions:**

The collection of CSF into EDTA tubes does not influence the result of the standard CSF analysis. However, a presumed positive effect of EDTA on cell preservation could not be shown in the present study.

## Background

Cerebrospinal fluid (CSF) analysis is pivotal in the diagnostic work-up of patients with central nervous system (CNS) signs, and CSF should be collected and analysed whenever an infectious or inflammatory disease, neoplasia or degenerative disorder of the brain or spinal cord is suspected [1, 2]. Because of its immediate physical relation to the CNS, CSF offers a more accurate reflection of any on-going pathology in the brain, spinal cord and meninges than blood samples [1–4].

A standard CSF analysis includes a total nucleated cell (TNC) count, a protein concentration measurement, a cytological evaluation and differential cell count [2]. In healthy dogs with normal CSF findings, the TNC count should be less than five cells/µL, and the protein content should not exceed 30 mg/dL for CSF collected from the cerebellomedullary cistern and 45 mg/dL for caudal lumbar samples [2, 5]. The TNC count and protein concentration can be elevated particularly in the presence of infectious or inflammatory CNS disease, due to an increase in permeability of the blood brain barrier. The differential cell count is typically also shifted in these cases [2, 6]. A cytological CSF examination is essential even in the presence of a normal TNC count, as abnormalities in cell types and structure can indicate CNS disease [2, 7, 8].

Cerebrospinal fluid samples may be collected into either ethylenediaminetetraacetic acid (EDTA) or plain tubes. EDTA is primarily used for haematological examinations due to its anticoagulant effect [9]. It has, however, been proposed that any fluid sample undergoing cytological evaluation should be placed in an appropriate amount of EDTA [10]. This is in order to reduce the effect of possible blood contamination, as cell aggregation can affect the cell counts and also the cell morphology. EDTA stabilised CSF may therefore be superior when evaluating total cell counts, morphology and differential cell counts. However, it is commonly stated that the EDTA content causes a false elevation in the total protein concentration and that it dilutes the sample, thereby affecting the diagnostic interpretation [11, 12]. Plain tubes are therefore traditionally recommended for the collection of CSF in dogs [2, 11, 12]. To the authors’ knowledge, however, there are no validated studies supporting this practice.

The purpose of this study was to determine if the choice of tube (EDTA or plain) influences the outcome of the standard CSF analysis in terms of protein concentration, cell count, and cell morphology. We hypothesized that there was no difference in the protein concentration or cell count in CSF samples collected in EDTA versus plain tubes, and that the preservation of cell morphology was better in EDTA stabilised samples.

## Methods

### Animals and samples

The study was completed at the University Hospital for Companion Animals, University of Copenhagen between February and July 2016. CSF samples were collected prospectively from dogs presenting to the University Hospital’s Neurology Service for diagnostic purposes only. In addition, samples were collected from dogs presenting to the hospital for euthanasia for varying reasons, in order to obtain a larger sample size within the study period.

Samples were only included if sufficient volumes for both EDTA and plain tubes were available and if the samples could be processed and analysed within 30 min of collection. Written owner consent was collected in all cases. The study was approved by the Local Administrative and Ethics Committee, Department of Veterinary Clinical Sciences, University of Copenhagen.

### CSF collection and analysis

Cerebrospinal fluid samples were obtained from the cerebellomedullary cistern upon aseptic preparation of the puncture site in all dogs. In dogs presenting for diagnostic purposes, CSF was collected with the dog placed in lateral recumbency under general anaesthesia. Pre-medication was chosen based on the clinical status of the dog, and in all dogs, anaesthesia was maintained on either isoflurane or sevoflurane. In dogs that presented for euthanasia, samples were collected immediately after death had occurred. CSF was collected in both EDTA (BD, NJ, USA) and plain plastic tubes (Frisenette, Knebel, Denmark), EDTA before plain tubes. Each tube was filled with 0.5–1 mL of CSF, taking the dog’s size into account. All samples were analysed within 30 min of collection. The CSF analysis was undertaken according to the laboratory’s standard protocol and included a macroscopic evaluation (colour and viscosity), a semi-quantitative protein concentration measurement, manual red blood cell (RBC) and TNC count, and a differential cell count.

The protein concentration in each CSF sample was determined using urinary dipsticks (Multistix^®^ 10 SG, Siemens, Erlangen, Germany), which is considered a valid screening method for measurement of protein concentration in CSF in dogs [13]. Ten microliter of CSF were applied to the protein field on the dipstick, and the result was read 60 s after the application. The dipstick was read by a technician. The technician was not blinded as to whether the CSF came from EDTA or plain tubes, but was blinded to the purpose of the study. The protein concentration was categorized as negative (0 mg/dL), trace (< 30 mg/dL), 1+ (30–100 mg/dL), 2+ (100–300 mg/dL), 3+ (300–2000 mg/dL) or 4+ (> 2000 mg/dL).

To ensure a homogenic cell distribution, CSF samples were turned on a Rotator SB3 (Stuart, Stone, UK) for 2 to 4 min before further processing. Manual RBC and TNC counts were performed using a Neubauer improved (Superior Marienfeld, Lauda-Königshofen, Germany) or a Bürker-Türk (Scherf Präzision, Meiningen, Germany) haemocytometer. The cell counts were evaluated by a technician. The technician was not blinded as to whether the CSF came from EDTA or plain tubes, but was blinded to the purpose of the study. Ten microliter aliquots of undiluted CSF were placed onto each side of the haemocytometer chamber. The cells were allowed to settle for 2 min in a humidified environment, before being placed under the microscope. RBC and TNC counts were determined by counting all cells in the centre square and four corner squares in both chambers. The total number of cells counted, equalled the number of cells/μL. If the cell count deviated more than 10% when comparing the two chambers, the procedure was repeated.

Cytocentrifuged preparations were made for differential cell counts. Two-hundred microliter of CSF were transferred with a pipette to a cytocassette, which was placed in the cytocentrifuge (StatSpin Cytofuge 2^®^, Beckman Coulter, GA, USA) along with a counterweight. The samples were centrifuged at 1300 rpm (93×*g*) for 8 min. The slides were manually stained with Hemacolor^®^ (Merck KGaA, Darmstadt, Germany). A differential nucleated cell count was performed on 200 cells or all cells, if fewer than 200 cells were present on the slides. Leukocytes were classified as polymorphonuclear (neutrophils and eosinophils), lymphocytes or monocytes/macrophages.

The preservation of cell morphology was subjectively evaluated as satisfactory or unsatisfactory. As no clear guidelines exist for quantifying the quality of cell morphology, strict guidelines were defined for the purpose of the present study. The slide was evaluated as satisfactory if less than 25% of the cells on the slide were destroyed or unidentifiable, whereas the sample was evaluated as unsatisfactory if more than 25% of the cells were destroyed or unidentifiable. A cell was considered as destroyed or unidentifiable if the nucleus or cell membrane was not intact. Morphological scores were assigned by one of the authors (MBMN) with vast experience with cytological evaluations. This evaluation was blinded, with the assessor not knowing if the preparations were made from CSF from EDTA or plain plastic tubes.

### Statistical analysis

Based on results of the D’Agostino-Pearson normality test, data was not considered consistent with a Gaussian distribution. For descriptive statistics of variables pertaining to CSF, median, interquartile range and total range were reported. A Chi square test for trend was used to examine the difference in the semi-quantitatively measured protein concentration between CSF samples in EDTA and plain tubes. When comparing RBC, TNC and differential cell counts between CSF in EDTA and plain tubes, a Wilcoxon matched pair’s test was used. For evaluating the difference between satisfactory and unsatisfactory cell morphology on slides made from EDTA stabilised CSF and CSF from plain tubes, a Fisher’s exact test was used. A P-value of < 0.05 was considered statistically significant. Statistical analyses were performed using GraphPad Prism 7.

## Results

Thirty-two paired CSF samples were included in the study, 14 samples from dogs with neurological diseases and 18 samples from dogs being euthanized for various reasons.

The group of dogs that presented for a neurological work-up included three Labrador retrievers, two large mixed breed dogs and one of each of the following: Beagle, Border terrier, Cavalier King Charles spaniel, Cocker spaniel, French bulldog, Lhasa apso, a medium mixed breed, Standard poodle and Welsh Springer spaniel. Five dogs were diagnosed with idiopathic epilepsy, three dogs with intervertebral degenerative disc disease, one with idiopathic facial paresis, one with Lafora disease, one with steroid responsive meningitis arteritis, one with degenerative myelopathy, one with suspected structural epilepsy and one with idiopathic myoclonic episodes.

The group of dogs that presented for euthanasia included four Labrador retrievers, three Golden retrievers, two German shepherd dogs and one of each of the following: Bichon havanais, Border collie, Boxer, Cavalier King Charles spaniel, Danish spitz, Danish Swedish farm dog, Flat coated retriever, Rottweiler and Shih tzu. Six dogs were euthanized due to behavioural problems, four due to neoplastic disease, four due to degenerative joint disease, two dogs due to old age, one due to idiopathic chylothorax and one dog due to a chronic dermatological problem.

There was no statistically significant difference in the semi-quantitative protein concentration measurement when comparing CSF samples from EDTA and plain plastic tubes (P > 0.99).

The median manual TNC count was 2/μL (Q1–Q3: 1–8/µL, range 0–1765/µL) from CSF samples in EDTA and 2/μL (Q1–Q3: 1–7/µL, range 0–1758/µL) from CSF in plain tubes. There was no statistically significant difference in the median TNC count between CSF in EDTA and plain tubes (P = 0.85).

The median manual RBC count was 3/μL (Q1–Q3: 0–7.5/µL, range 0–257/µL) from CSF samples in EDTA and 1/μL (Q1–Q3: 0–7.5/µL, range 0–103/µL) from CSF in plain tubes. There was no statistically significant difference in the median RBC count between CSF in EDTA and plain tubes (P = 0.18). Table 1 gives an overview over the 32 CSF samples and the analysis from EDTA and plain tubes with regards to manual RBC and TNC counts and also the semi-quantitatively measured protein concentrations.

**Table 1:** Results of the cerebrospinal fluid analysis from EDTA and plain plastic tubes

Dog ID	TNC count		RBC count		Semi-quantitative protein concentration	
	EDTA	Plain	EDTA	Plain	EDTA	Plain
1	3	1	118	20	Trace	Trace
2	1	1	0	1	Trace	Trace
3	2	1	257	19	Trace	Trace
4	7	4	8	8	2+	2+
5	4	1	3	0	1+	1+
6	37	35	3	1	1+	1+
7	1	1	1	0	Trace	Trace
8	0	0	0	1	Negative	Negative
9	1765	1758	135	103	2+	2+
10	2	0	6	35	1+	1+
11	2	2	94	22	Trace	Trace
12	14	11	3	1	1+	1+
13	1	2	0	2	Trace	Trace
14	0	1	4	17	Trace	Trace
15	2	3	0	0	Trace	Trace
16	0	1	5	0	Trace	Trace
17	32	25	11	6	Trace	Trace
18	2	2	3	0	1+	1+
19	3	2	0	0	Trace	Trace
20	0	1	3	0	Trace	Trace
21	79	65	141	15	1+	1+
22	1	0	3	2	Trace	Trace
23	1	1	3	2	Trace	Trace
24	1	2	0	0	Trace	Trace
25	0	1	1	0	Trace	Trace
26	15	16	3	0	Trace	Trace
27	1	2	0	0	Trace	Trace
28	8	7	0	0	Trace	Trace
29	0	0	10	2	Trace	Trace
30	15	12	1	3	Trace	Trace
31	2	0	0	0	Trace	Trace
32	3	7	4	1	Trace	Trace

The semi-quantitatively measured protein concentration was categorized as negative (0 mg/dL), trace (< 30 mg/dL), 1+ (30–100 mg/dL), 2+ (100–300 mg/dL), 3+ (300–2000 mg/dL) or 4+ (> 2000 mg/dL)

*EDTA* ethylenediaminetetraacetic acid, *RBC* red blood cells, *TNC* total nucleated cells

There was no statistically significant difference in the proportionate differential cell count from CSF in EDTA and plain tubes when looking at polymorphonucleated cells (P = 0.9), lymphocytes (P = 0.84) and monocytes/macrophages (P = 0.86). The results from the statistical analysis of differential cell counts from CSF in EDTA and plain tubes are presented in Table 2.

**Table 2:** Results of the statistical analysis of differential cell counts from EDTA and plain tubes

Analyte	EDTA			Plain			P-value
	Median	IQR	Min–max	Median	IQR	Min–max	
Polymorphonuclear cells (%)	8	0–20	0–66	7	0–20	0–69	0.9
Lymphocytes (%)	40	12.3–63.8	0–97	45	11.8–66.5	0–93	0.84
Monocytes/macrophages (%)	31	0–49.5	0–100	28.5	0–47.3	0–100	0.86

*EDTA* ethylenediaminetetraacetic acid, *IQR* interquartile range

In 13 of 32 cytological preparations (40.6%) made from EDTA stabilised CSF, the cell morphology was evaluated as satisfactory according to the quality assessment standards defined for the present study. When looking at preparations made from CSF from plain plastic tubes the cell morphology in 16 of 32 preparations (50%) was evaluated as satisfactory. There was no statistically significant difference in the quality of cell morphology between preparations from EDTA and plain plastic tubes (P = 0.45).

## Discussion

According to the findings in this study, the collection of CSF into EDTA tubes does not influence the result of the standard CSF analysis in terms of the semi-quantitatively measured protein concentrations, TNC counts and differential cell counts. It should, however, be noted that CSF should be collected into an appropriate transport media or sterile tube if culturing of the fluid is anticipated [10].

It is commonly stated that EDTA will contribute to a false high protein concentration, thus potentially altering the interpretation of the CSF analysis [2, 11, 12]. However, as EDTA is an aminopolycarboxylic acid [9] and not a protein, the EDTA content should not contribute to the measurement of either albumin or globulin. This could be confirmed in the present study, where we found no difference in the semi-quantitatively measured protein concentrations in CSF collected into EDTA and plain tubes. The findings in this study suggest that the EDTA content in commercially available tubes does not influence the protein concentration in canine CSF and that EDTA tubes can be used for CSF collection and analysis if indicated. In humans, plain plastic tubes are traditionally used for CSF collection in order to avoid a potential binding effect of collection tube additives [3, 4]. EDTA stabilised CSF is, however, used when measuring proteins and peptides such as cytokines and DNA, which are subject to a high degree of enzymatic degradation in vitro [3, 4].

Another concern commonly expressed when using EDTA tubes for CSF sampling is that the EDTA content will dilute the CSF sample, thereby falsely lowering the TNC count [2, 3, 11, 12]. In the present study, we used EDTA sample tubes for a volume of 1 mL and between 0.5 and 1 mL of CSF was collected into each tube, meaning that the tubes were not always correctly filled. Yet, as cell counts in the present study did not differ significantly between the two different tubes, this concern does not seem to be substantial. Paediatric EDTA tubes are, however, available and can be used if small sample sizes are going to be collected, thereby avoiding the concern of potentially diluting the CSF sample.

Two of the CSF samples showed differences in the TNC count that could be clinically relevant. In one sample the TNC count was seven cells/µL in CSF from the EDTA tube and four cells/µL in CSF from the plain tube, this sample was taken from a dog diagnosed with idiopathic epilepsy. The second sample was collected from a dog euthanized due to non-CNS related neoplastic disease. In this sample the TNC count was three cells/µL in CSF from the EDTA tube and seven cells/µL in CSF from the plain tube. These findings are most likely incidental and not dependant on the choice of collection tube. The findings, however, emphasise the importance of using information from signalment, history, physical and neurological examination and imaging studies for proper interpretation of CSF changes for accurate diagnosis in the individual case [14, 15].

The majority of CSF samples (68.8%) included in the study were considered normal based on a TNC count under five cells/µL. This may complicate extrapolation to CSF samples with higher cell counts. We, however, believe that the distribution of sample cell counts reflects the picture we see clinically where many dogs with neurological diseases have normal TNC counts. Another limitation in this study is that the dipstick readings and cell counts were not blinded, even though the technicians performing the tests were blinded to the purpose of the study. More reliable results would be achieved with the dipstick being read by a machine or performing quantitative protein measurements.

In our cytological evaluations, each Hemacolor^®^ stained slide was assigned a morphology score (satisfactory or unsatisfactory) to try and quantify cell preservation, and we hypothesised that slides made from EDTA stabilised CSF samples would be superior with regards to the preservation of cell morphology. Surprisingly, this hypothesis could not be confirmed as we found no significant difference in the quality of cell morphology between the two groups. A possible explanation for this may be that all cytological preparations, both plain and EDTA stabilised, were made within 30 min of CSF collection. It is well known that cells in CSF degenerate quickly, possibly due to the hypotonicity and the low protein content of CSF, and that CSF samples therefore should be processed as soon as possible after collection, as in this study [16–18]. Although the anticoagulant effect of EDTA should be immediate in blood and other fluid samples [9, 10, 19], it may be difficult to demonstrate the superiority of EDTA stabilised samples with regards to preservation of cell morphology within 30 min of collection, as the effect of the natural cell degradation may not be evident at this early point in time. It has previously been shown that the leukocyte concentrations in plain CSF deteriorate in a time dependent fashion. Statistically significant changes are, however, first evident 2 h after CSF collection [16]. This suggests that a positive effect of EDTA on cell preservation in the present study may have been demonstrated in samples stored for 2 h or more, rather than 30 min before processing.

Accordingly, EDTA stabilised CSF may be superior to plain CSF if the sample needs to be stored before an analysis can take place. Several reports recommend altering the CSF sample when it cannot be analysed as soon as possible after collection [16, 18]. The addition of foetal calf serum or hetastarch to canine CSF has been shown to improve the stability of the CSF sample [16]. These additives may, however, not be readily available at the veterinary clinic as opposed to EDTA tubes. The preservative effect of EDTA over time on CSF from dogs has not yet been investigated. Unfortunately, in this study there was not enough CSF volume available from dogs to perform serial cytological investigations over time, and so the question remains unanswered at this point. In human medicine, adding EDTA to synovial fluid has shown to improve leukocyte stability for up to 48 h [20, 21], supporting the idea that EDTA stabilised CSF samples may be superior to plain, if analysis cannot be performed in-house shortly after collection.

In this study a semi-quantitative protein concentration measurement technique was chosen, using urine reagent strips. This has previously been reported to be a valid initial screening method to estimate the CSF protein concentration [13]. The method is however highly specific for albumin detection and less specific for globulin detection and therefore only provides a crude quantification of protein in the CSF sample [13]. We can therefore not exclude that the results in this study would be different with a quantitative protein measurement technique. However, protein measurements did not differ within any of the paired observations, supporting the use of urine reagent strips as an easily accessible, low-cost method, which is not influenced by EDTA as a stabilising agent.

## Conclusion

The collection of CSF into EDTA tubes did not influence the result of the standard CSF analysis, neither with regards to the semi-quantitatively measured protein concentration nor TNC count. A presumed positive effect of EDTA on cell preservation could however not be shown. The potential role of EDTA as an additive to prevent leukocyte degeneration over time remains elusive.
